# Role and Molecular Mechanisms of Pericytes in Regulation of Leukocyte Diapedesis in Inflamed Tissues

**DOI:** 10.1155/2019/4123605

**Published:** 2019-05-07

**Authors:** Paulina Rudziak, Christopher G. Ellis, Paulina M. Kowalewska

**Affiliations:** Department of Medical Biophysics, Robarts Research Institute, Schulich School of Medicine & Dentistry, University of Western Ontario, London, ON, Canada

## Abstract

Leukocyte recruitment is a hallmark of the inflammatory response. Migrating leukocytes breach the endothelium along with the vascular basement membrane and associated pericytes. While much is known about leukocyte-endothelial cell interactions, the mechanisms and role of pericytes in extravasation are poorly understood and the classical paradigm of leukocyte recruitment in the microvasculature seldom adequately discusses the involvement of pericytes. Emerging evidence shows that pericytes are essential players in the regulation of leukocyte extravasation in addition to their functions in blood vessel formation and blood-brain barrier maintenance. Junctions between venular endothelial cells are closely aligned with extracellular matrix protein low expression regions (LERs) in the basement membrane, which in turn are aligned with gaps between pericytes. This forms preferential paths for leukocyte extravasation. Breaching of the layer formed by pericytes and the basement membrane entails remodelling of LERs, leukocyte-pericyte adhesion, crawling of leukocytes on pericyte processes, and enlargement of gaps between pericytes to form channels for migrating leukocytes. Furthermore, inflamed arteriolar and capillary pericytes induce chemotactic migration of leukocytes that exit postcapillary venules, and through direct pericyte-leukocyte contact, they induce efficient interstitial migration to enhance the immunosurveillance capacity of leukocytes. Given their role as regulators of leukocyte extravasation, proper pericyte function is imperative in inflammatory disease contexts such as diabetic retinopathy and sepsis. This review summarizes research on the molecular mechanisms by which pericytes mediate leukocyte diapedesis in inflamed tissues.

## 1. Introduction

Recruitment of leukocytes to sites of infection or injury is a tightly regulated multistep process controlled by leukocyte interactions with the endothelial layer and the ability of the leukocyte to breach the vascular wall. The complete process of leukocyte penetration of the vascular wall is referred to as diapedesis. Most of leukocyte-endothelial interactions are observed in venular compartments [1–4]. However, exceptions exist in highly specialized vascular beds where substantial numbers of adherent leukocytes are observed in vessels other than venules, such as hepatic sinusoids [5], pulmonary capillaries [6], and arterioles in the heart [7]. The initial step described in the classical paradigm of leukocyte recruitment in venules is leukocyte tethering to and rolling on the endothelium. These transient adhesive interactions are mediated by endothelial P-selectin/CD62P and E-selectin/CD62E binding to leukocyte glycoprotein ligands such as P-selectin glycoprotein ligand- (PSGL-) 1/CD162 and E-selectin ligand- (ESL-) 1. The rolling leukocyte stops as it adheres to endothelial cells, an interaction mediated by adhesion molecules expressed on the endothelium, such as intercellular adhesion molecule- (ICAM-) 1/CD54 and vascular cell adhesion molecule- (VCAM-) 1/CD106. These molecules interact with leukocyte *β*_2_ (lymphocyte function-associated antigen- (LFA-) 1; CD11a/CD18) and *α*_4_ integrins, respectively [8]. Next, leukocytes may crawl for variable distances on the endothelium via ICAM-1 and macrophage-1 antigen (Mac-1; CD11b/CD18)-dependent mechanism [9]. This step is followed by leukocyte breaching of the endothelial layer, which mostly happens in a paracellular fashion. This process is mediated by adhesion molecules on endothelial cells—platelet-endothelial cell adhesion molecule- (PECAM-) 1/CD31, junctional adhesion molecule- (JAM-) A, ICAM-2/CD102, and endothelial cell-selective adhesion molecule (ESAM) [10–12]. The extravascular leukocyte then migrates in the interstitium along a chemokine gradient.

Vascular walls of precapillary arterioles, capillaries, and postcapillary venules are composed of an endothelial layer, pericytes, and a basement membrane associated with both cell types. Pericytes are contractile cells with long processes wrapping around vessels. Pericytes are present at intervals along the walls of capillaries and venules with variable morphologies. Density of pericytes in vasculature varies in different types of vessels and tissues. Pericyte-to-endothelial cell ratios in humans can range from 1 : 1 in the retina, 1 : 10 in the lung, and 1 : 12.4 (±7.1) in the dermis [13, 14]. Pericytes are mainly identified by the expression of *α*-smooth muscle actin, neural/glial antigen 2 (NG2), platelet-derived growth factor receptor- (PDGFR-) *β*, nestin, desmin, CD146, and FOXD1 progeny [15–21]. The role of pericytes in blood flow regulation in different tissues is still debated. Interestingly, in the brain, pericytes were shown to contribute to postischemic injury by reducing capillary red blood cell flow through their persistent contraction around capillaries [22].

Pericytes are involved in fine-tuning inflammatory responses by controlling leukocyte diapedesis. Pericyte-mediated leukocyte diapedesis involves crawling through the basement membrane, adhesion to and crawling on pericytes, and finally migration through pericyte gaps into interstitial space towards inflammatory foci [23]. Before migrating through gaps between pericytes, neutrophils first interact with adhesion molecules on the surface of pericytes [24]. After stimulation with cytokines, pericytes express higher levels of proinflammatory molecules, such as major histocompatibility complexes (MHC II), and are able to increase the phagocytic activity of neutrophils [25]. This review will further describe specific cellular and molecular mechanisms of these leukocyte-pericyte interactions with an emphasis on murine neutrophils and monocytes—the cells that are the focus of most studies on this topic.

## 2. Pericytes Mediate Leukocyte Diapedesis

Leukocyte migration through the vascular wall is commonly observed to happen along specific paths, with several leukocytes exiting through the same area. These paths are characterized by regions with lower levels of extracellular matrix proteins in the venular basement membrane, specifically laminin 8, laminin 10, collagen IV, and nidogen [24, 26]. These areas are termed low expression regions (LERs). LERs are closely aligned with endothelial cell junctions and pericyte gaps in small venules of murine cremaster muscle, forming the aforementioned preferential paths for leukocyte migration [24]. Specifically, the majority of neutrophils were associated with endothelial cell junctions (mostly at tricellular junctions), and over 75% of neutrophils were aligned with LERs and pericyte gaps in interleukin-1*β*- (IL-1*β*-) treated cremaster venules [24]. Neutrophils were not observed to go through pericyte bodies and only migrated through pericyte gaps. This suggests that pericyte position influences the route of leukocyte migration through the endothelial layer. LERs that were aligned with pericyte gaps were found in the basement membrane of venules of various organs in mice, including the cremaster muscle, skin, mesentery, peritoneal wall, and diaphragm [26]. The quantity and size of LERs varied widely between the different tissues, with trends indicating that smaller venules had smaller LERs but in greater numbers [26].

Neutrophils express integrin receptors, such as *α*_2_*β*_1_ and *α*_6_*β*_1_, for collagen IV and laminin 10 in the vascular membrane [24]. Neutrophil migration through LERs in laminin 10, laminin 8, and type IV collagen networks in response to various inflammatory mediators, such as leukotriene B4 (LTB4), CXCL1/keratinocyte chemoattractant (KC), tumor necrosis factor- (TNF-) *α*, and endotoxin as well as ischemia/reperfusion injury, was shown to cause the LERs to expand in cremaster muscle venules [26]. The same study also confirmed that neutrophils induce LER enlargement in mouse skin after intradermal injection of TNF-*α*. A portion of neutrophils that transmigrated through the LERs of the cremaster venules in response to topical LTB4, or intrascrotal injection of CXCL1, TNF-*α*, or endotoxin, was observed to carry cleaved laminin 10 and laminin 8 fragments on their surface but no collagen IV fragments were detected [26]. This may be a potential mechanism of LER remodelling characterized by enlargement and thinning of these regions [26, 27]. The serine protease neutrophil elastase on the surface of transmigrating cells [28] contributed to the observed LER remodelling [24]. Interestingly, monocytes did not appear to remodel the LERs as the neutrophils did, suggesting that leukocyte-specific mechanisms for migration through LERs exist [26]. Moreover, monocytes do not enlarge LERs because they are of small diameter, are more deformable than neutrophils, and form narrower protrusions, allowing them to squeeze through LERs of the venular basement membrane in CCL2/monocyte chemoattractant protein- (MCP-) 1-stimulated cremaster muscle [29].

LER enlargement also happens in concert with pericyte gap enlargement. Confocal microscopy of cremaster postcapillary venules of *αSMA-RFPcherry × Lys-EGFP-ki* mice with fluorescent pericytes and leukocytes provided insight into how neutrophils interact with pericyte gaps to breach the pericyte layer [30]. The authors showed that the expression and activation of TNF-*α* and IL-1*β* receptors on pericytes increase their expression of ICAM-1 and CXCL1 and that pericyte gaps enlarge in response to stimulation by TNF-*α* or IL-1*β*. The pericyte-derived CXCL1 induced neutrophil crawling on pericyte processes *in vivo* until the neutrophils reached gaps between adjacent pericytes. This crawling behaviour was mediated by ICAM-1 interactions with Mac-1 and LFA-1 and was a prerequisite for neutrophils to breach the pericyte layer [30]. Furthermore, neutrophils were shown to preferentially migrate through gaps that were 8–50 *μ*m in size between two pericytes and that were rich in CXCL1 and ICAM-1 expression at the gap border. Several leukocytes were shown to exit through a given gap. The first cell to breach a gap moved slower and with a more torturous path than subsequent migrating cells. Also, the leading leukocyte appeared more hesitant by inserting a protrusion through a gap multiple times at different sites before finally migrating through it. Of note, the authors did not observe any pericyte transcellular migration [30].

The ultrastructural details behind neutrophil and pericyte contacts were observed using scanning force microscopy [23]. TNF-*α*-stimulated porcine pericytes expressed ICAM-1 and interacted with neutrophils through cellular extensions that both cell types formed. Pericyte extensions were generally broader and larger than neutrophil extensions. However, after neutrophils interacted with pericytes, neutrophil extensions grew to almost the same height and width as the pericyte extensions [23]. While Proebstl et al. found that pericyte gap enlargement in inflammatory conditions was independent of interactions with neutrophils [30], another study reported that contact between neutrophils and pericytes causes gap enlargement in IL-1*β*-stimulated cremasteric venules as well as pericyte relaxation in culture [27]. Pericyte relaxation and gap expansion allowed for increased neutrophil transmigration. Treatment of mice with neutralizing antibodies to ICAM-1 or *α*_6_ integrin (laminin receptor) halted neutrophils in the basement membrane, which prevented neutrophil-pericyte contact, and consequently, there was no gap or LER enlargement [27]. The authors also showed that the neutrophil contact-induced pericyte relaxation occurred via decrease in RhoA/ROCK pathway signaling which led to the loss of stress fibers and focal adhesions in pericytes *in vitro* [27]. Pericyte relaxation also appears to be mediated by endothelial macrophage migration inhibitory factor (MIF). Mice with conditional knockout of endothelial cell MIF that were treated intranasally with lipopolysaccharide (LPS) had significantly decreased leukocyte infiltration in the lung interstitial space, alveoli, and bronchoalveolar lavage fluid, which was associated with decreased venular pericyte relaxation [31].

**Figure 1:**
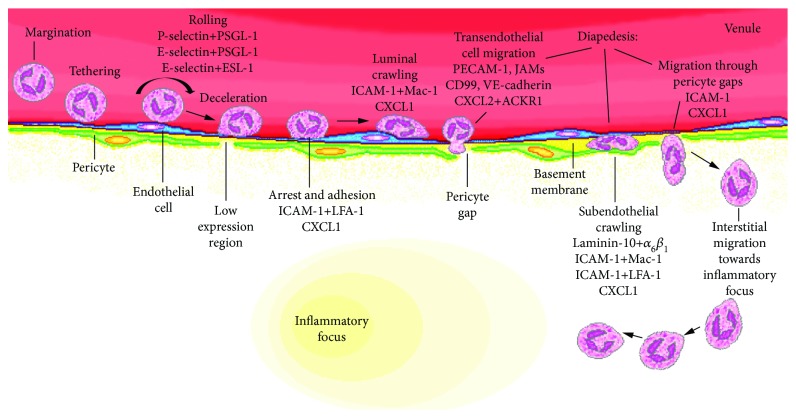
Role of pericytes in leukocyte diapedesis in inflammation. Major adhesion molecules and chemokines in the neutrophil recruitment cascade in mouse cremasteric venules are depicted. Leukocytes marginate towards the vessel wall and tether to inflamed venular endothelium via interactions of selectins with leukocyte carbohydrate ligands. Rolling leukocytes slow down and firmly adhere to the endothelium through binding of intercellular adhesion molecules to leukocyte integrins. Leukocytes crawl along endothelial cells to find a suitable spot for extravasation. After movement through the endothelial layer, leukocytes move through the basement membrane and crawl on pericyte processes towards preferential pericyte gaps. Leukocyte contact with pericytes causes enlargement of these gaps along with the associated low expression regions in laminins and collagen IV. Leukocytes exit through pericyte gaps and migrate under a chemotactic gradient in the interstitium towards inflammatory foci. PSGL-1: P-selectin glycoprotein ligand-1; ESL-1: E-selectin ligand-1; ICAM-1: intercellular adhesion molecule-1; LFA-1: lymphocyte function-associated antigen-1; Mac-1: macrophage-1 antigen; PECAM-1: platelet-endothelial cell adhesion molecule-1; JAMs: junctional adhesion molecules; VE-cadherin: vascular endothelial cadherin; ACKR1: atypical chemokine receptor-1; MIF: macrophage migration inhibitory factor.

Pericytes contribute to a mechanism involving temporal and spatial distribution of chemokines across the venular wall, which ensures unidirectional luminal-to-abluminal neutrophil migration [32]. Mice deficient in CXCR2 (chemokine receptor for CXCL1 and CXCL2/macrophage inflammatory protein- (MIP-) 2) and mice treated with neutralizing antibodies to CXCL1 and CXCL2 have blunted neutrophil transmigration in cremaster venules after local stimulation with TNF-*α*. Specifically, blockade of CXCL1 inhibited neutrophil-endothelial adhesion and intraluminal crawling while blockade of CXCL2 blunted leukocyte transendothelial migration. Neutrophil crawling on pericytes was also abrogated by the neutralization of CXCL1, and consequently, the neutrophils accumulated in the venular wall. Thus, the sequential effects of CXCL1 and CXCL2 and then again CXCL1 are necessary for successful leukocyte diapedesis [32]. The endothelial cells were the source of luminal CXCL1, and pericytes were the source of abluminal CXCL1. Neutrophils produced CXCL2 and deposited the chemokine on the atypical chemokine receptor 1 (ACKR1) which is enriched at endothelial cell junctions. Endothelial ACKR1 was necessary for depositing CXCL2 at endothelial cell junctions, which was essential for luminal-to-abluminal neutrophil migration.  Figure 1 outlines generalized steps of pericyte-mediated leukocyte extravasation derived from studies on the cremaster muscle.

Intriguingly, venular pericytes are not the only ones that interact with and influence leukocytes during inflammation. Stark et al. delineated a role for arteriolar and capillary pericytes in the murine skin microcirculation in potentiating leukocyte responses to inflammatory stimuli using intravital two-photon microscopy of NG2-DsRed-CX_3_CR1-GFP and NG2-DsRed-LysM-eGFP mice [33]. The authors showed that arteriolar and capillary pericytes increase expression of ICAM-1 and secrete MIF in response to subcutaneous injection of TNF. The authors also showed that human placental pericytes trigger chemotaxis of neutrophils and monocytes via MIF. *In vivo*, MIF secretion induced the chemotactic migration of neutrophils and monocytes that exited inflamed postcapillary venules, leading to contact with capillary and arteriolar pericytes via ICAM-1. Specifically, pericytes formed pathways along capillaries and arterioles for rapid migration of extravasated leukocytes. Blocking ICAM-1 resulted in lower frequency, velocity, and duration of pericyte-leukocyte interactions [33]. The pericyte-myeloid cell interaction made the interstitial migration of neutrophils and macrophages towards inflammatory foci more efficient after subcutaneous injection of the bacterial peptide N-formylmethionyl-leucyl-phenylalanine. The interaction also increased their immunosurveillance capacity in addition to stimulating these myeloid cells to express activation markers, adhesion molecules, and Toll-like receptors (TLRs) [33].

## 3. Effects of Inflammatory Mediators on Pericyte Interactions with Leukocytes

The effects of various proinflammatory molecules on pericyte morphology and behaviour in relation to leukocyte responses are summarised as follows.

### 3.1. Damage-Associated Molecular Patterns (DAMPs) and Pathogen-Associated Molecular Patterns (PAMPs)

DAMPs and PAMPs are conserved molecular patterns recognized by pattern recognition receptors, including TLRs, which initiate a rapid innate immune response. PAMPs refer to common molecular motifs on microorganisms while DAMPs are endogenous molecules released or exposed by damaged or dying cells in the host. DAMPs and PAMPs trigger the expression of adhesion molecules and chemokines on the surface of endothelial cells, which promotes neutrophil migration to sites of tissue damage or infection [33]. Stimulation of pericytes with LPS and incubation with DAMPs induced pericytes to express chemoattractants, such as CXCL1, CXCL8/IL-8, and CCL2 as well as MIF and IL-6 [33, 34]. These chemoattractants efficiently induced the chemotaxis of monocytes and neutrophils.

### 3.2. TNF-*α*

TNF-*α* affects pericyte expression of integrins which in turn affects pericyte interactions with components of the extracellular matrix [35]. Pericytes express *α*_1_*β*_1_ and *α*_2_*β*_1_ integrins, which bind collagen. Pericytes also express *α*_5_ and *α*_6_ integrins, which bind fibronectin and laminin, respectively. Pericytes particularly express high levels of *α*_5_ integrin and lower levels of *α*_1_, *α*_2_, and *α*_6_ integrins. Adhesion assays showed that pericytes strongly attach to fibronectin and collagen I and IV, weakly attached to laminin-1, and do not attach to heparan sulfate proteoglycan [35]. Fibronectin and collagen I promote remodelling of pericyte shape. Function-blocking antibodies showed that *α*_2_ integrin is necessary for adhesion of pericytes to collagen. In culture, TNF-*α* caused pericytes to change their expression of integrins from *α*_1_ to *α*_2_ to stimulate pericyte proliferation and change in pericyte morphology and motility [35]. Also, stimulation of human brain pericytes with TNF-*α* increased expression of VCAM-1, resulting in a threefold increase in T cell adhesion to pericytes [36, 37].

### 3.3. IL-1*β*

IL-1*β* enhances pericyte secretion of sVCAM-1, CX_3_CL1 (fractalkine), CCL2, and IL-6 [38]. IL-1*β*, LPS, and TNF-*α* are potent inducers of CXCL8 secretion by pericytes, which chemoattracts neutrophils to pericytes in culture. In coculture of primary porcine brain capillary endothelial cells and primary porcine brain capillary pericytes, these inflammatory mediators induced neutrophil transmigration. This process was supported by metalloproteinase-9 (MMP-9) [25]. Specifically, MMP-9 released neutrophils that adhered to pericytes, and inhibition of MMP-9 enhanced adhesion of neutrophils to pericytes.

### 3.4. IFN-*γ*

Interferon-*γ*- (IFN-*γ*-) stimulated pericytes do not secrete CXCL8 and do not increase transendothelial neutrophil migration when compared to non-IFN-*γ*-stimulated pericytes [25]. Also, IFN-*γ* stimulation of pericytes reduced activation of T cell receptors through cell-to-cell contact, and pericytes in long-term cultures highly express T cell inhibitors program death ligands 1 and 2 (PD-L1 and PD-L2) when stimulated with IFN-*γ* [39]. Moreover, IFN-*γ*-treated MHC II pericytes did not stimulate allogeneic CD4 T cell proliferation or cytokine production, indicating that pericytes are poorly immunogenic [40]. These studies suggest that IFN-*γ* skews pericytes towards an immunosuppressive phenotype.

### 3.5. IL-17

IL-17 is mainly secreted by T helper 17 cells but is also produced by other types of T cells, natural killer cells, type 3 innate lymphocytes, and particular subtypes of neutrophils. IL-17 has two receptor types, IL-17RA and IL-17RC. Pericytes are more responsive to IL-17 stimulation compared to human umbilical and dermal endothelial cells, and all three express low levels of IL-17RC [41]. IL-17 increased both mRNA and protein expression of IL-6 and CXCL8 by pericytes. Pericytes activated with IL-17 had increased expression of proinflammatory factors, including granulocyte colony-stimulating factor, granulocyte-macrophage colony-stimulating factor, CXCL1, CXCL6, and CXCL8. When TNF-*α* was added with IL-17, pericytes had a greater increase in expression of IL-6, CXCL8, CXCL5, and CCL20 transcripts, highlighting the synergistic effects of IL-17 and TNF-*α* on pericytes. Moreover, IL-17-stimulated pericytes caused neutrophils to synthesize TNF-*α*, IL-1*α*, IL-1*β*, and CXCL8. In general, IL-17-stimulated pericytes enhance neutrophil activation, cytokine production, survival, and phagocytic capacity compared with basal pericytes [41]. Thus, IL-17 promotes pericyte modulation of neutrophil function in inflammation.

## 4. Signaling Pathways in Pericytes during Leukocyte Extravasation

Inhibition of RhoA/ROCK signaling pathways in pericytes induces pericyte relaxation, creating larger gaps between pericytes [27]. Pericytes relax when neutrophils form direct contacts with them, causing pericytes to become elongated and narrower with increased motility. Transwell insert approach was used to observe transmigration of neutrophils through a single layer of pericytes [27]. In the same study, pericytes were treated with drugs that either activate or deactivate RhoA signaling pathway to change pericyte monolayer permeability. Norepinephrine was used to activate the RhoA pathway, which maintains stress fibers and focal adhesions, and causes contraction of pericytes. Norepinephrine decreased pericyte gap area along with the ability of leukocytes to penetrate these gaps. Before penetration, neutrophils migrate through the space between endothelial cells and pericytes. Norepinephrine increased the retention of neutrophils in this space. On the other hand, Tolazoline, which inhibits RhoA activity, significantly enlarged pericyte gaps and LERs, ultimately increasing leukocyte extravasation [27]. LPS-stimulated pericytes also induce the MyD88 pathway, and inhibition of the MyD88 pathway caused a 6-fold decrease in neutrophil recruitment and reduction in the amount of activated proinflammatory macrophages in the mouse kidney [34].

## 5. Pericytes in Disease

### 5.1. T Cells and the Tumor Microenvironment

Pericytes generally support leukocyte transmigration into interstitial tissue but not CD4 T cell recruitment in the tumor microenvironment. Tumor-derived vascular pericytes induce CD4 T cell inactivation/dysfunction, which is thought to influence immune surveillance during continuous tumor growth [42]. This was demonstrated using isolated CD4 T cells from ovalbumin- (OVA-) immunized mice with pericytes incubated in the presence of OVA, where ICAM-1 expression on tumor-derived pericytes engaged LFA-1 on CD4 T cells to induce anergy. Mechanisms underlying the effects of pericytes on T cells have been examined using human cultured cells. Human placental MHC II^−^ (untreated) pericytes and MHC II^+^ (treated with IFN- *γ*) pericytes cultured across semipermeable membranes from allogeneic endothelial cell/CD4 T cell cocultures consistently inhibited CD4 T cell proliferation [40]. However, addition of neutralizing antibodies to pericytes against either IL-10, transforming growth factor-*β* (TGF-*β*), or both significantly relieved the inhibition of CD4 T cell production caused by MHC II^+^ pericytes. Furthermore, human pericytes have high expression of PD-L1 and PD-L2 under stimulation of IFN- *γ* [39]. They do not induce activation or generation of allogeneic resting T cells without IFN-*γ* and instead increase the frequency of allogeneic CD25^high^FoxP3^+^ T regulatory cells. Addition of TGF-*β*1 to human brain pericytes can also reduce gene expression of chemokines and adhesion molecules, specifically CXCL8, CX_3_CL1, CCL2, and VCAM-1 [38]. Pericytes were observed to contribute to immunosuppression in human malignant glioma environment where they negatively correlated with leukocyte recruitment and infiltration of tumors by CD8 T cells in particular [43]. Conversely, in another clinical study, pericytes contributed to the production of CXCL9, which formed heterocomplexes with CXCL12 in brain biopsies. These chemokines attracted CD8 effector T cells and malignant B cells into the primary central nervous system lymphoma in human tissues, which is a histological feature of the tumor [44]. Pericytes also influence metastasis, but this topic is beyond the scope of this review and is discussed elsewhere [45].

### 5.2. Diabetes

The retina has the largest pericyte density in the human body. Loss of retinal pericytes is a symptom of early-stage diabetic retinopathy in patients and animal studies. In T cell function assays, mouse and human retinal pericytes inhibited T cell responses by expressing PD-L1 through direct cell-to-cell contact with T cells [46]. This mechanism protected retinal endothelial cells from T cell-mediated apoptosis. However, under hyperglycemic media culture conditions, retinal pericytes are less immunosuppressive towards T cells. Thus, reduced immunosuppression by pericytes may contribute to the chronic and uncontrolled retinal inflammation in diabetic patients [46]. PDGF*β* is involved in the recruitment of pericytes, and PDGF*β*^+/-^ mice with diabetic retinopathy had decreased pericyte density and developed microvascular lesions in the retina [47]. High glucose conditions can cause an imbalance between proapoptotic and prosurvival factors in human retinal pericytes. Human retinal pericytes cultured in intermittent high glucose and/or hypoxic conditions had an increase in proapoptotic molecules including Fas ligand (FasL), active caspase-8, tBid, p53, and Bax [48]. FasL binds to Fas receptor to activate intracellular factors, which causes activation of caspase-8 and pericyte apoptosis. Interestingly, in a diabetic mouse model, recruitment of leukocytes via ICAM-1/*β*_2_ integrin interactions in the retinal microvasculature led to a loss of endothelial cells and pericytes, resulting in an increased number of acellular capillaries [49].

It is important to note that high glucose-induced activation or dysfunction of pericytes can be suppressed/prevented by ascorbic acid (vitamin C) treatment. Recent studies indicate that ascorbic acid effectively prevents pericyte apoptosis induced by high glucose concentration [50, 51]. Ascorbic acid is sequestered in pericytes, potentially allowing pericytes to provide a small supply of vitamin C to neighbouring endothelial cells [50, 51]. Human brain pericytes cultured in glucose for six days with ascorbate showed that high glucose in both long- and short-term cultures depleted intracellular ascorbate in pericytes and increased pericyte apoptosis [50]. However, daily addition of 50 or 100 *μ*M ascorbate decreased apoptosis of pericytes cultured under basal and high glucose concentration. Pericytes were also cultured in high levels of glucose for six to seven days to determine if increased apoptosis in high glucose-induced pericytes is caused by receptor for advanced glycation end products (RAGE) activation. Addition of a RAGE inhibitor prevented apoptosis in high glucose-treated pericytes [50]. In addition, although pericytes used the ascorbate transporter SVCT2 to take up ascorbic acid, there was net efflux of ascorbate [51]. The authors suggested that ascorbate efflux from pericytes has the potential to act as an important mechanism for supplying vitamin C to neighbouring endothelial cells when extracellular concentration of ascorbate is decreased under oxidative stress [51].

### 5.3. Sepsis (Endotoxemia)

Pericytes are not merely passive mural cells but active participants in the inflammatory response. LPS-stimulated pericytes express TLR4 [52]. Using RT-PCR approach, it has been shown that stimulation of rat microvascular lung pericytes with LPS for 4 h results in an increase in mRNA of CD14, TLR2, and TLR4 expression that is maintained up to 18 h [52]. Activation of pericyte TLR4 elicits the production of various cytokines. Specifically, LPS-stimulated pericytes displayed nuclear accumulation of NF-*κ*B and increased protein expression of cytokines and chemokines—CXCL8, IL-6, CXCL1, CCL2, CXCL2, and CXCL3—and adhesion molecules such as ICAM-1 and VCAM-1 [53]. The increased expression of ICAM-1 and VCAM-1 on pericytes resulted in increased peripheral blood leukocyte adhesion to the pericyte monolayer. These *in vitro* experiments support the notion that pericytes have pathophysiological implications in sepsis.

Maintaining pericyte attachment in the microvascular network is crucial to prevent vascular leakage and excessive leukocyte recruitment in sepsis. To study the basis of pericyte loss in sepsis, mice were treated with LPS. LPS decreased the expression of sirtuin 3 (Sirt3), hypoxia inducible factor (HIF-2*α*), and Notch3 in the lungs [54]. LPS treatment also reduced the density of pericytes, increased angiogenin 2 (Ang-2), and decreased Ang-1 and tyrosine-protein kinase receptor (Tie-2) expression. Knockout of Sirt3 increased expression of Ang-2 but decreased expression of Tie-2, HIF-2*α*, and Notch3, resulting in an overall loss of pericyte coverage and an increase in vascular leakage 24 h after LPS treatment. However, overexpression of Sirt3 reduced Ang-2 expression and increased Ang-1, Tie-2, HIF-2*α*, and Notch3 expression in LPS-treated mice, which attenuated LPS-induced pericyte loss and vascular leakage. Interestingly, a reduction in pericyte density was accompanied by increased neutrophil/macrophage CD11b^+^ infiltration in both the heart and lung after LPS treatment [54]. The neutrophil/macrophage CD11b^+^ infiltration was further exacerbated by Sirt3 knockout, while overexpression of Sirt3 reduced the number of infiltrating cells. The authors believe that pericyte loss promotes leukocyte infiltration in tissues. Another study also found that intraperitoneal LPS treatment of mice caused disruption of the blood-brain barrier by causing disorganization of the basal lamina and pericyte detachment 6–24 h after injection [55]. This outcome was further compounded by the increase in microglial activation characterized by enlarged cytoplasm and cell bodies, irregular shape, and enhanced microglia Iba-1 staining [55]. Thus, pericytes may be a potential therapeutic target given the significance of pericyte integrity in diseases like septic encephalopathy.

## 6. Conclusion and Future Perspectives

The role of pericytes in inflammation has been traditionally underappreciated. Pericytes act as sensors and regulators of inflammatory processes in the microvasculature with an important impact on leukocyte function [33]. Pericytes not only support leukocytes during diapedesis; they also potentiate leukocyte effector functions in the interstitial space. Pericyte gaps maintain the integrity of the basement membrane by forming preferential paths for leukocyte recruitment in addition to forming a barrier to excessive leukocyte extravasation [24]. Thus, the role of pericytes in diseases that are driven by chronic inflammation should be further investigated. For example, in animal models of peritoneal dialysis, the peritoneal catheter implant was found to significantly contribute to pathological peritoneal membrane alterations, namely fibrosis and new vessel formation [56]. These vessels were found to have significantly increased levels of leukocyte-endothelial cell interactions and increased numbers of extravascular leukocytes. Given that loss of pericytes on venules leads to increased leukocyte infiltration, it is possible that there is either a lack of pericytes or presence of dysfunctional pericytes on the immature venules that form in the peritoneal lining, allowing for excess leukocyte extravasation and constant inflammation in the peritoneum that ultimately leads to dialysis technique failure. Also, the regenerated microvasculature in the mouse extensor digitorum longus muscle after ischemia-induced obliteration is characterized by abnormal architecture and microvascular dysfunction, manifested as altered red blood cell dynamics and inadequate oxygenation [57]. Further investigation is warranted into whether venules of the regenerated microvasculature have aberrant pericyte coverage or function. The altered pericyte density or function may lead to increased leukocyte infiltration in the steady state, contributing to ongoing inflammation that prevents the microvasculature from working properly. Lastly, microvascular dysfunction is a hallmark of sepsis [58, 59]. One of the ways in which microvascular dysfunction is manifested in a rat model of septic skeletal muscle microcirculation is intermittent and stopped flow of red blood cells through the capillaries. It would be important to establish whether capillary pericyte constriction contributes to stopped-flow capillaries of the skeletal muscle in the early stage of sepsis.

Given their role in inflammation, pericytes are potential targets for anti-inflammatory therapies. Pericytes suppress T cell reactions in the retina, which protects retinal endothelial cells from inflammation-mediated apoptosis. However, this immunosuppressive ability of retinal pericytes is lost under hyperglycemic conditions. Under septic conditions, the functionality and density of pericytes in capillary networks are altered, causing increased vascular leakage and interactions with leukocytes.

Further studies are necessary to investigate how pericytes mediate extravasation of specific leukocyte subtypes other than neutrophils to sites of inflammation and how they may be mutually influenced by perivascular macrophages and mast cells. Also, whether pericytes regulate reverse migration of leukocytes across the vascular wall remains to be investigated along with the molecular mechanisms of this poorly understood process [60]. Most studies on the interactions of pericytes and leukocytes used *in vitro* approaches and require further confirmation *in vivo* with intravital video microscopy techniques. In particular, studies are needed to address inflammatory function(s) of pericytes in animal disease models and more work needs to be done on human cells/tissues in order to increase clinical relevance of pericyte function in inflammation.
